# Clinical validation of C_12_FDG as a marker associated with senescence and osteoarthritic phenotypes

**DOI:** 10.1111/acel.14113

**Published:** 2024-05-06

**Authors:** William S. Hambright, Victoria R. Duke, Adam D. Goff, Alex W. Goff, Lucas T. Minas, Heidi Kloser, Xueqin Gao, Charles Huard, Ping Guo, Aiping Lu, John Mitchell, Michael Mullen, Charles Su, Tamara Tchkonia, Jair M. Espindola Netto, Paul D. Robbins, Laura J. Niedernhofer, James L. Kirkland, Chelsea S. Bahney, Marc Philippon, Johnny Huard

**Affiliations:** ^1^ Center for Regenerative Sports Medicine Steadman Philippon Research Institute Vail Colorado USA; ^2^ Department of Physiology and Biomedical Engineering Mayo Clinic Rochester Minnesota USA; ^3^ Department of Biochemistry and Molecular Biology and Biophysics, Institute on the Biology of Aging and Metabolism University of Minnesota Minneapolis Minnesota USA; ^4^ Division of General Internal Medicine, Department of Medicine Mayo Clinic Rochester Minnesota USA; ^5^ Orthopaedic Trauma Institute University of California San Francisco San Francisco California USA; ^6^ The Steadman Clinic Vail Colorado USA

**Keywords:** aging, cell senescence, osteoarthritis, senolytics

## Abstract

Chronic conditions associated with aging have proven difficult to prevent or treat. Senescence is a cell fate defined by loss of proliferative capacity and the development of a pro‐inflammatory senescence‐associated secretory phenotype comprised of cytokines/chemokines, proteases, and other factors that promotes age‐related diseases. Specifically, an increase in senescent peripheral blood mononuclear cells (PBMCs), including T cells, is associated with conditions like frailty, rheumatoid arthritis, and bone loss. However, it is unknown if the percentage of senescent PBMCs associated with age‐associated orthopedic decline could be used for potential diagnostic or prognostic use in orthopedics. Here, we report senescent cell detection using the fluorescent compound C_12_FDG to quantify PBMCs senescence across a large cohort of healthy and osteoarthritic patients. There is an increase in the percent of circulating C_12_FDG^+^ PBMCs that is commensurate with increases in age and senescence‐related serum biomarkers. Interestingly, C_12_FDG^+^ PBMCs and T cells also were found to be elevated in patients with mild to moderate osteoarthritis, a progressive joint disease that is strongly associated with inflammation. The percent of C_12_FDG^+^ PBMCs and age‐related serum biomarkers were decreased in a small subgroup of study participants taking the senolytic drug fisetin. These results demonstrate quantifiable measurements in a large group of participants that could create a composite score of healthy aging sensitive enough to detect changes following senolytic therapy and may predict age‐related orthopedic decline. Detection of peripheral senescence in PBMCs and subsets using C_12_FDG may be clinically useful for quantifying cellular senescence and determining how and if it plays a pathological role in osteoarthritic progression.

## Graphical abstract

This study reports on senescence phenotyping across a large cohort of healthy and osteoarthritic patients. We found an increase in the percent of circulating C_12_FDG^+^ PBMCs commensurate with increases in age and senescence‐related serum biomarkers. Interestingly, we further found the senescence phenotype to be elevated in patients with mild to moderate osteoarthritis, a progressive joint disease that is strongly associated with inflammation.

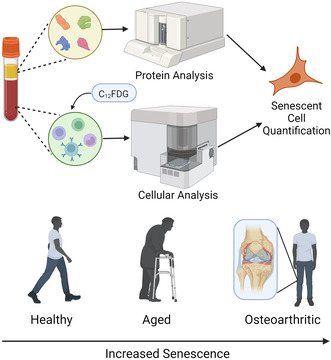

**Abbreviations**
BH: Benjamini‐Hochberg
C12FDG: 5‐Dodecanoylaminofluorescein Di‐β‐D‐Galactopyranoside
CBC: complete blod count
FDR: false discovery rate
FMO: fluorescence minus one
OA: osteoarthritis
PBMCs: peripheral blood mononuclear cells
SASP: senescence‐associated secretory phenotype
SA‐β‐gal: senescence‐associated β‐galactosidase
uPAR: urokinase plasminogen activator receptor

## 1 INTRODUCTION

Aging is the greatest risk factor for most of the chronic diseases and conditions that limit health span, including cancers, cardiovascular diseases, diabetes, osteoporosis, osteoarthritis, and neurodegenerative disorders (Yousefzadeh et al., 2021). Despite these strong links to human disease, our understanding of the pathophysiology of aging remains fairly limited. Cellular senescence was first described in the 1960s by Hayflick, who reported on the limited replicative potential of normal cultured human fibroblasts, and is now recognized to be a key biologic driver of aging (Hayflick, 1965). Senescence is considered to be a tumor‐suppressor mechanism that is activated in response to oncogene activation to suppress uncontrolled growth. However, senescence is also driven by different types of stress, resulting in cell cycle arrest and a secretory phenotype that is pro‐inflammatory and tissue‐damaging (Tchkonia & Kirkland, 2018; Xu et al., 2018). The deleterious effects of an increased senescent cell burden with age were demonstrated by the observed delay or attenuation of many age‐related diseases, suppression of frailty, and extended healthspan following either the genetic or pharmacologic‐mediated elimination of senescent cells (Chaib et al., 2022; Tchkonia & Kirkland, 2018; Xu et al., 2018).

The majority of studies on senescence have been performed in vitro or in small animal models. A recent systematic review of senescence in human tissue samples found that the heart, liver, and respiratory systems were the most investigated, with most studies showing higher expression of senescence markers from diseased tissue compared to controls (Tuttle et al., 2021). However, there are only a few reports evaluating the detection of senescent cells from peripheral blood of human patients, which is of increased clinical utility due to the ease of minimally invasive blood sampling (Liu et al., 2009; Tuttle et al., 2021). Various types of peripheral blood mononuclear cells (PBMCs) can undergo senescence in culture, however, the dynamics of senescence in PBMCs in vivo and their potential relationships with age‐related decline and disease are poorly understood (Pangrazzi & Weinberger, 2020). Senescence‐associated β‐galactosidase (SA‐β‐gal) activity is an established and widely used marker for cellular senescence and has allowed for the detection of senescent cells by flow cytometry by staining with the florescent X‐gal substrate C_12_FDG (Cahu & Sola, 2013; Noppe et al., 2009). C_12_FDG is a lipophilic compound that when hydrolyzed by β‐galactosidase produces a fluorogenic signal detectable by flow cytometry. Within PBMCs, T cells are known to exhibit senescence signatures with increasing age, leading to declines in immunogenicity, accumulation of highly differentiated T‐cell subsets, and a diminished receptor repertoire, with decreased output from bone marrow lymphoid cells and thymic involution that are thought to contribute to the inflammaging phenotype (Pangrazzi & Weinberger, 2020). Importantly, others have reported an increased signal in CD8^+^ T cells with age that is commensurate with the expression of senescence‐related pathways (Martinez‐Zamudio et al., 2021). Thus, the quantitative detection of senescent, or senescent‐like, PBMCs using fluorogenic SA‐β‐gal compounds would allow for analysis of senescence dynamics with chronological age and identify potential therapeutic opportunities to modulate peripheral senescent cell burden in diseases of advanced age.

Importantly, clinically relevant detection modalities for senescence may contribute to a mechanistic understanding of the pathophysiology of age‐related diseases. Osteoarthritis (OA) is a progressive joint disease resulting in cartilage degeneration, pain, and loss of knee function for which there are no FDA‐approved therapies that treat the root cause. OA is typically diagnosed by a history of joint pain associated with movement coupled with radiographic loss of articular cartilage (Glyn‐Jones et al., 2015). Various putative biomarkers have been characterized as potential indicators of structural, biochemical, or physiological severity of OA pathogenesis (Glyn‐Jones et al., 2015). However, there are currently no reliable biomarkers that are diagnostic or prognostic signatures of OA (Glyn‐Jones et al., 2015). Cellular senescence and the release of pro‐inflammatory cytokines/chemokines, proteases, and other factors collectively referred to as senescence‐associated secretory phenotype (SASP) (Coppe et al., 2008; Tchkonia & Kirkland, 2018) have been described for their ability to induce OA (Xu et al., 2017). Peripheral senescent T cells have also been linked to conditions such as frailty, rheumatoid arthritis, and bone loss (Covre et al., 2020). Thus, the ability to accurately detect and/or track peripheral senescent cells may help to measure a key pathological contributor to OA and potentially assist in deciding interventional strategies.

A key interest of our group is investigating the contribution of cellular senescence to age‐associated musculoskeletal decline and understanding peripheral dynamics of senescent PBMC subsets associated with orthopedic conditions including OA. The goals of the current study were to evaluate whether senescent cells can be detected in the peripheral blood of a large cohort of human subjects, determine how this population of cells changes with age and in the setting of a chronic disease such as OA, and understand whether the over‐the‐counter supplement fisetin results in a decrease in the number of C_12_FDG^+^ cells. We hypothesized that human C_12_FDG^+^ PBMCs would increase with chronological age, be associated with senescence biomarkers, become elevated in patients with OA, and be reduced in patients taking fisetin.

## 2 MATERIALS AND METHODS

### 2.1 Study participants

The protocols and policies for sample collection and enrollment were approved by the Vail Health IRB for healthy participants (#2019‐58) and mild to moderate OA participants (#2019‐16, NCT04210986). Healthy participants consisted of 266 individuals enrolled between December 2019 through March 2022 between the ages of 18–85 years. For cell senescence correlations with chronological age, 126 participants were eliminated from analysis due to confounding medical histories or concomitant senolytic therapy. Data from fisetin takers were constructed as a subgroup analysis from the healthy participant cohort where individuals self‐reported fisetin dosage at consistent doses (100 mg/day) and similar frequencies. In order to capture random fisetin takers at similar doses and frequencies with similar collection times, we were limited in the number of participants to evaluate. OA patients between the ages of 40–85 years were ambulatory and graded as mild to moderate (KL II‐IV) with baseline NRS pain scores of 4–10 and on stable baseline medication doses for ≥2 months (*n* = 75). Patients were excluded if they had clinically significant co‐existing conditions, diabetes, abnormal heart conditions, arthroscopy of the target knee or hip within 2 years, planned arthroscopy of the knee or hip during the study period, intra‐articular treatment within 16 weeks prior to screening, regenerative joint procedures, and any active or suspected autoimmune disease. To find further information on the inclusion and exclusion criteria of the healthy and OA patients, please visit clinicaltrials.gov.

### 2.2 Venipuncture procedure

Blood samples were collected from each subject at up to three separate timepoints using a standard venipuncture procedure. 30 mL of venous peripheral blood were drawn into a syringe prefilled with 5 mL of ACD‐A. 0.5 mL of this sample was transferred into a microcentrifuge tube to obtain a Complete Blood Count (CBC) using a Cell Dyn Hematology analyzer. An additional 5 mL of peripheral blood were drawn into a 5 mL syringe and transferred to a red cap vacutainer tube without anticoagulant for downstream serum isolation.

### 2.3 Total PBMC collection and T‐cell enrichment

Within 2 h of whole blood collection, PBMCs were isolated from peripheral blood. T cells were enriched using RosetteSep™ Human T‐Cell Enrichment Cocktail (StemCell Technologies, Cat #15061) according to the manufacturer's protocol. Both PBMCs and T cells were collected via SepMate™ collection tubes (StemCell Technologies, Cat #85460), using Lymphoprep™ (StemCell Technologies, Cat #7861) density gradient, and washed twice. All isolated cells were frozen in CryoStor® (StemCell Technologies, Cat #7930) at a controlled rate using Mr. Frosty containers.

### 2.4 Serum collection

Red capped vacutainer tubes were spun at 1500 *g* for 15 min at room temperature then the acellular serum layer was collected. Three 400 μL aliquots of serum were transferred into sterile microcentrifuge tubes, then stored at −80°C until batch processing for biomarker quantification.

### 2.5 C_12_FDG staining and flow cytometry

Frozen human PBMCs were rapidly thawed in a water bath and immediately diluted (4:1) in FACS buffer (PBS, 5% FBS, and 5 mM EDTA Bio‐Rad Cat #: 1610729). Cells were centrifuged 300 *g* for 5 min and counted to ensure a minimum of 2.0 × 10^5^ cells per tube when resuspended in 1 mL culture medium (DMEM/F12 Thermo Fisher Scientific, Cat #: 11‐320‐033, with 10% FBS and 1% pen/strep Thermo Fisher Scientific Cat #: 15140148). Fresh PBMCs and TCs were resuspended in 1 mL culture media and bafilomycin (Cell Signaling Technology Cat #: 54645) (100 nM) were added. Cells were incubated on a shaker (150 rpm) in 5% CO_2_ at 37°C for 1 h. Each sample was then divided in two—one stained with C_12_FDG (Abcam Cat #: ab273642) (6.5 μM) and the other an unstained control—and incubated on a shaker (150 rpm) at 5% CO_2_ 37°C for 1 h. All fractions were diluted (2:1) with FACS buffer, centrifuged 500 *g* for 5 min, washed with PBS at 500 *g* for 5 min, then resuspended in 100 μL FACS buffer. 5 μL Human Seroblock (Bio‐Rad, Cat #: BUF070B) was added to all samples for 10 min at room temperature. Samples were transferred to ice and stained with remaining antibodies and incubated at 4°C for 30 min (assay‐specific, either CD87 only, or CD87, CD3, and CD14) (Table 1). Cells were washed at 500 *g* for 5 min and resuspended in FACS buffer. Just before running each tube, Propidium Iodide (PI) (0.63 μL) was added as a viability stain. The multi‐color assay was run on Cytek® Northern Lights (16v, 14b) cytometer. The single‐color TCs and PBMCs assay were run on a Guava® easyCyte™ HT (Guava®) flow cytometer in triplicate.

**TABLE 1:** Fluorescent antibodies and stains.

Specificity (CD)	Fluorochrome	Vendor	Catalog	Purpose	Volume per 100 μL
					(μL)
PI	Propidium Iodide	Thermo Fisher	BMS500PI	Live/Dead	0.63 per 300 μL
3	PE	Thermo Fisher	MA1‐19624	T cells	0.63
14	Alexa Flour 532	Thermo Fisher	58‐0149‐41	Monocytes	2.5
C_12_FDG	C_12_FDG	Abcam	138777‐25‐0	Senescence	6.5 μM
87	PerCP‐eFlour710	Thermo Fisher	46‐3879‐42	Senescence	2.5

The instrument settings for the Cytek Cytometer were set at: Threshold 500,000 PBMCs‐ Cytek assay settings, FSC 35, SSC 199, SSC‐B 197 and threshold 300,000 SF‐ Cytek assay settings, FSC 50, SSC 300, SSC‐B 580. Voltages were not adjusted from Cytek assay settings. Prior to running the full panel each antibody/stain was titrated with a seven‐fold dilution and optimal concentration was determined by inflection point on a stain index curve. Single stains and fluorescence minus one (FMO) controls were established for each fluorochrome. Single stains were used for unmixing purposes and FMO's were used for setting up gates. Cells were gated by first selecting for the cellular cluster using SSC and FSC. Cells were interrogated for high autofluorescence using SSC (violet) H and SSC‐B‐H. Cells were selected for singlets with both FSC and SSC discrimination. Singlets were selected for viable cells. Cell populations of interest (all cells, TCs (CD3^+^, CD14^−^), and Monocytes (CD14^+^, CD3^−^)) were gated from viable cells. Each population was interrogated for senescence using C_12_FDG and CD87.

### 2.6 Multiplex analysis of serum biomarkers

Serum supernatant fractions were thawed at room temperature and centrifuged at 1500 *g* for 10 min to remove remaining debris. Biomarkers in each sample were quantified using multiplex assays performed with MILLIPLEX® MAP magnetic bead panels according to the manufacturer's protocol (Table 2). All samples were run in duplicate, and plates were read on a Luminex® 100/200™ platform using xPonent® software (EMD Millipore Corp). Analyte concentrations were calculated using Belysa™ Immunoassay Curve Fitting Software (EMD Millipore Corp) and included in resulting analysis that provided >70% reliability.

**TABLE 2:** Multiplex analytes and associated kits (EMD Millipore Corp.).

Kit name (#cat)	Abbreviated name	Name
TIMP panel 1 (#HTMP1MAG‐54K‐02)	TIMP‐1	Tissue inhibitor matrix metalloproteinase‐1
	TIMP‐2	Tissue inhibitor matrix metalloproteinase‐2
MMP panel 1 (#HMMP1MAG‐55K‐03)	MMP‐3	Matrix metalloproteinase‐3
	MMP‐12	Matrix metalloproteinase‐12
MMP panel 2 (#HMMP2MAG‐55K‐04)	MMP‐1	Matrix metalloproteinase‐1
	MMP‐2	Matrix metalloproteinase‐2
	MMP‐9	Matrix metalloproteinase‐9
TGF‐β universal kit (#TGFBMAG‐64K‐03)	TGF‐β1	Transforming growth factor‐beta 1
	TGF‐β2	Transforming growth factor‐beta 2
Human cytokine/chemokine multiplex panel (#HCYTOMAG‐60K‐13)	Eotaxin	N/A
	EGF	Endothelial growth factor
	GRO	Growth regulated oncogene
	IL‐15	Interleukin‐15
	IL‐1RA	Interleukin‐1 receptor antagonist
	IL‐8	Interleukin‐8
	IP‐10	Interferon‐gamma‐induced protein‐10
	MCP‐1	Monocyte chemoattractant protein‐1
	RANTES	Regulated upon activation, normal T cell expressed and presumably secreted chemokine
	TNF‐α	Tumor necrosis factor‐alpha
	PDGF‐AA	Platelet‐derived growth factor‐aa
	PDGF‐AB/BB	Platelet‐derived growth factor‐ab/bb
	IL‐6	Interleukin‐6
Human Bone Panel (#HBNMAG‐51K‐05)	Leptin	N/A
	OC	Osteocalcin
	SOST	Sclerostin
	PTH	Parathyroid hormone
	FGF‐23	Fibroblast growth factor‐23

### 2.7 Statistical analysis

Statistical analysis was performed using GraphPad Prism (Version 9.1). Descriptive statistics shown listed as mean ± standard deviation. Data were found to be non‐normally distributed through Kolmogorov–Smirnov normality testing. Associations among age, senescent cell burden, and biomarker data were thus calculated using nonparametric Spearman correlation testing. Continuous variables were compared using Mann–Whitney testing. Longitudinal differences within patients were determined using Wilcoxon matched pairs signed rank testing. *p*‐Values <0.05 (two‐tailed) were considered statistically significant and were adjusted for multiple comparisons using the Benjamini‐Hochberg (BH) procedure for false discovery rate (FDR) control at 0.25 level.

## 3 RESULTS

### 3.1 Flow cytometry‐based detection of senescent human PBMCs using C_12_FDG staining

**FIGURE 1:**
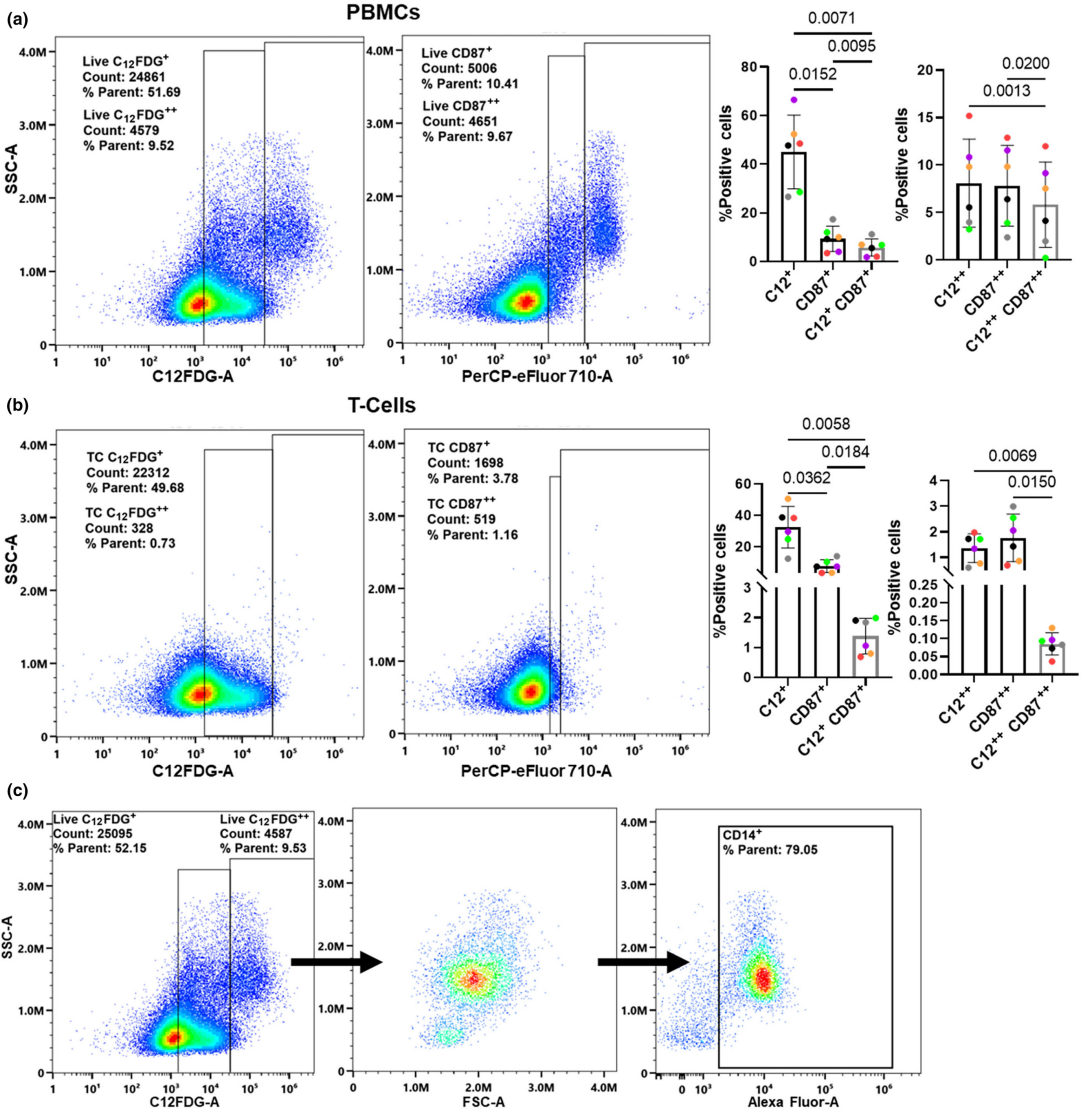
Detection of C_12_FDG senescent cells in human total PBMCs and T cells. (a) Comparative gating of live C_12_FDG and CD87 (uPAR) in total PBMCs. Quantification (right) of percent positive events per stain including co‐localization of C_12_FDG and CD87. (b) Comparative gating of live C_12_FDG^+^ and CD87^+^ (uPAR) in enriched CD3^+^ T cells. Quantification (right) of percent positive events per stain, including co‐localization of C_12_FDG^++^ (bright) and CD87^+^ cells. (c) Representative gating for CD3/CD14 co‐stained T cells enriched using RosetteSep showing 93% T‐cell purity (CD3^+^/CD14^−^) using the pan T‐cell marker CD3 and monocyte marker CD14. *Results were analyzed by ANOVA (*n* = 6).

C_12_FDG (5‐Dodecanoylaminofluorescein Di‐β‐D‐Galactopyranoside) is a lipophilic compound that fluoresces at a wavelength of 514 nm when hydrolyzed by β‐galactosidase, an enzyme encoded by the Glb1 gene upregulated during senescence. To identify potentially senescent C_12_FDG^+^ PBMCs, freshly isolated PBMCs were pre‐treated for 1 h with bafilomycin (100 nM) to alkalinize lysosomal β‐galactosidase and then stained with C_12_FDG (30 μM) for 1 h. The stained PBMCs were divided into C_12_FDG^++^ (bright) and C_12_FDG^+^ (dim) (Figure 1a). In addition, the C_12_FDG staining was reproducible using both fresh and from frozen buffy coat PBMCs (Figure S1). CD87 is a commonly reported cell‐surface senescence marker encoded by the *PLAUR* gene, which encodes the urokinase plasminogen activator receptor (uPAR) (Baart et al., 2020). *PLAUR* is upregulated in various cell types during senescence and is enriched in immune cells including neutrophils, monocytes, and macrophages (Baart et al., 2020). When co‐stained with an antibody against CD87, C_12_FDG^+^ PBMCs exhibited significantly higher percent of positive events than CD87^+^ events (Figure 1a). Interestingly, bright cell events led to nearly identical values for C_12_FDG^++^ and CD87^++^ (Figure 1a). To interrogate senescence signatures in PBMC subpopulations, T cells were enriched from total PBMCs and then similarly stained with CD87 and C_12_FDG (Figure 1b, Figure S2). The enrichment purity of T cells was confirmed to be >93% for CD3^+^/CD14^−^ T cells using traditional antibody staining (Figure 1c). As with total PBMCs, the percent of C_12_FDG^+^ T cells was significantly higher than CD87^+^ cells, but there remained strong alignment between C_12_FDG^++^ and CD87^++^ (Figure 1b). A significant decrease was noted in both PBMCs and T cells when calculating double positive C_12_FDG^++^/CD87^++^ compared to either of the singly‐stained cell populations (Figure 1a,b). These data suggest that C_12_FDG^++^ bright fraction of total PBMCs is aligned closely with CD87 staining and may thus be more reflective of physiological levels of senescence than C_12_FDG^+^ dim cells.

### 3.2 C_12_FDG^++^ PBMCs are associated with chronological age and are elevated in males

**FIGURE 2:**
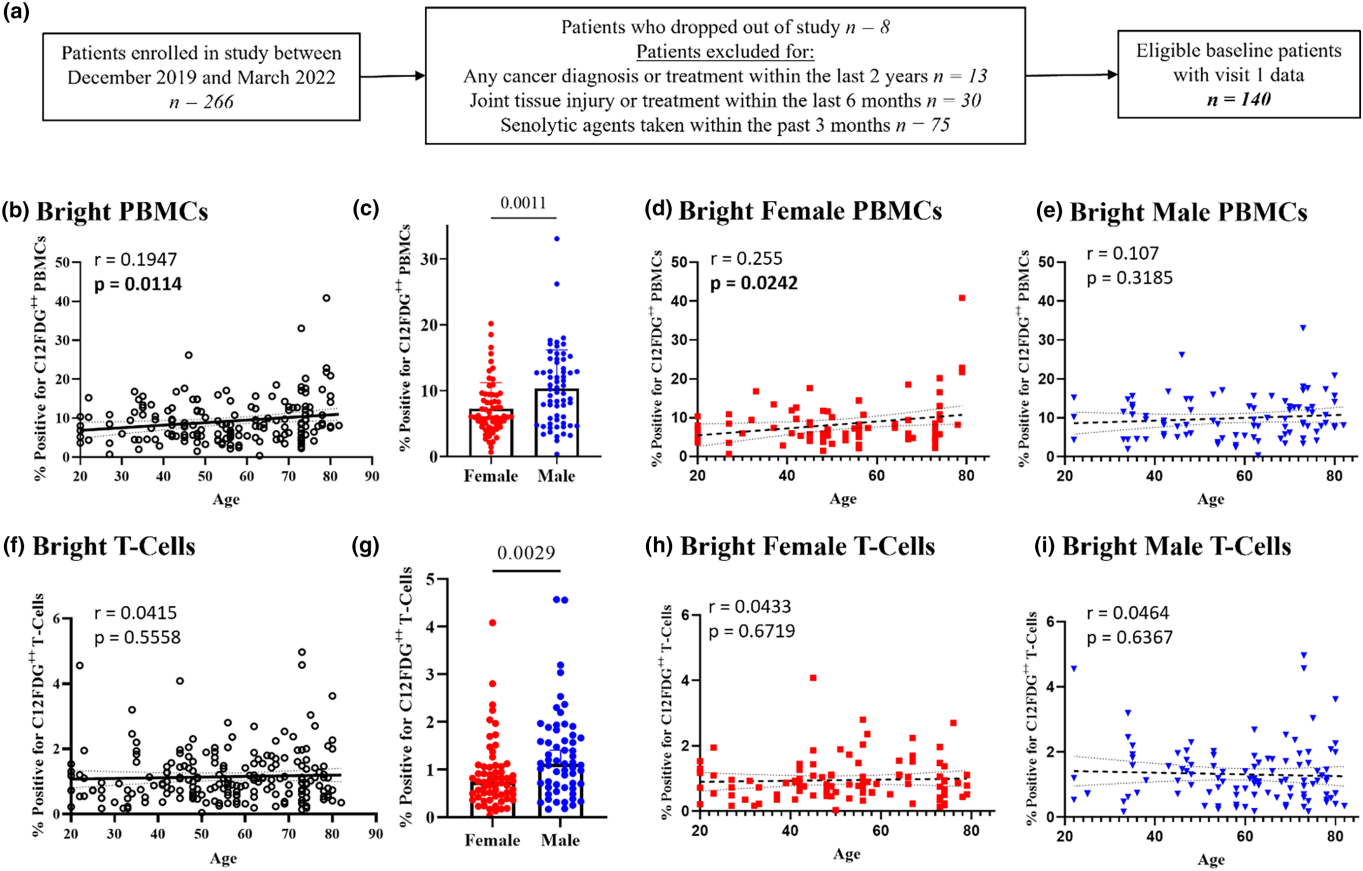
Percent C_12_FDG^++^ (bright) PBMCs increases with age and is elevated in age‐matched males compared to females. (a) Schematic of patient selection criteria and demographics (*n* = 168 visits). (b) Percent bright PBMCs significantly increases by 0.1947% per year of age and (c) were significantly elevated in males compared to females (*n* = 64/group, *p* = 0.0011). (d) Percent bright PBMCs of females significantly increases by 0.255% per year of age, while (e) percent bright PBMCs of males increases by 0.107% per year of age, but was not significantly correlated. (f) Bright TCs (*n* = 204 visits, *p* = 0.0029) were not significantly correlated with age but (g) were significantly elevated in males compared to females. (h, i) Percent bright TCs were not significantly correlated by either sex.

Accumulation of senescent cells in various tissues as well as in immune cell populations is related to biological aging. To assess whether C_12_FDG cells increased with chronological age, 266 human participants (age 18–85) were enrolled to measure senescence parameters in peripheral blood. After applying exclusion criteria to select generally healthy adults who were not taking any drugs with known senotherapeutic activity, a total of 140 human subjects were included for analysis (Figure 2a; age range 20–82, median age = 57). Consistent gating protocols were applied by a single blinded operator to define the C_12_FDG^++^ (bright) cells in PBMCs across this large cohort. The percent of C_12_FDG^++^ PBMCs significantly correlated with chronological age (Figure 2b; *r* = 0.1947; *p* = 0.0114) and were significantly elevated in healthy males versus females (Figure 2c; *p* = 0.0011). Interestingly, only within females was there a significant association with chronological age (Figure 2d, *r* = 0.255; *p* = 0.0242), whereas this was not significant within males (Figure 2e, *r* = 0.107; *p* = 0.3185).To examine C_12_FDG changes specifically in T cells, T cells were enriched from PBMCs prior to C_12_FDG staining as above (Figure 1c). However, although C_12_FDG^++^ T cells were significantly elevated in males (Figure 2g; *p* = 0.0029), no significant positive correlation was found with increasing age for C_12_FDG^++^ in our study cohort (Figure 2f; *r* = 0.04148; *p* = 0.5558). When segregated by sex, similarly, no significant correlation found with increasing age in females (Figure 2h, *r* = 0.0433; *p* = 0.6719) or males (Figure 2i, *r* = 0.0464; *p* = 0.6367) in percent of C_12_FDG^++^ T cells. The lack of a positive relationship between C_12_FDG^++^ T cells and age may be due to the relatively small percentage of C_12_FDG^++^ cells identified in this healthy patient cohort (1.14 ± 0.82%). Overall, the significant correlation in C_12_FDG^++^ PBMCs reinforces the utility of the C_12_FDG bright, but not dim, cell population as an indicator of peripheral senescence in immune cells known to exhibit age‐related decline.

### 3.3 C_12_FDG ^++^ PBMC's are correlated with serum biomarkers of aging and senescence

**FIGURE 3:**
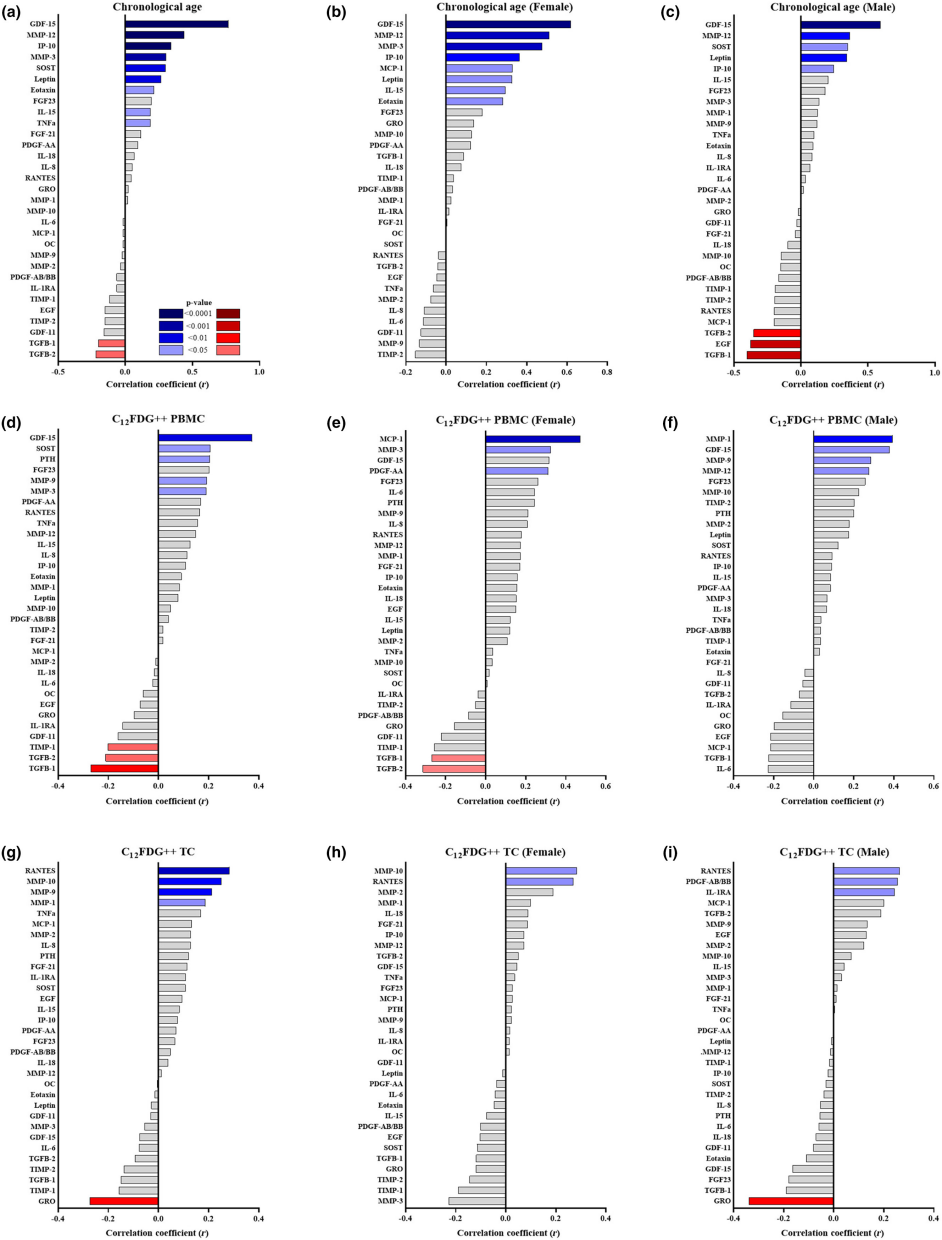
Association of C_12_FDG^++^ with chronological age and senescence‐related serum biomarkers. Correlation tree showing association of various serum analytes with chronological age for the (a) total population, (b) females and (c) males. Correlation tree showing association of percent C_12_FDG^++^ (bright) PBMCs with various serum analytes for the (d) total population, (e) females, and (f) males. Correlation tree showing association of percent C_12_FDG^++^ (bright) TCs with various serum analytes for the (g) total population, (h) females, and (i) males. FDR‐corrected two‐sided Spearman correlation with significant *p*‐values colored blue (positive correlation) and red (negative correlation) as indicated in the ledged (*n* = 144).

Given that we observed a significant correlation between C_12_FDG^++^ PBMCs and increasing age, we next evaluated the association between C_12_FDG^++^ PBMCs and serum levels of biomarkers related to aging and SASP in the study participants using multiplex immunoassays. Biomarkers found to positively correlate with increasing age included growth/differentiation factor‐15 (GDF‐15: *r* = 0.7678; *p* < 0.0001), interferon γ‐induced protein‐10 (IP‐10: *r* = 0.3401; *p* < 0.0001), matrix metalloprotease‐12 (MMP‐12: *r* = 0.44; *p* < 0.0001), MMP‐3 (*r* = 0.3035; *p* = 0.0009), sclerostin (SOST: *r* = 0.3002; *p* = 0.0030), leptin (*r* = 0.2654; *p* = 0.0023), eotaxin (*r* = 0.21210; *p* = 0.0122), interleukin‐15 (IL‐15: *r* = 0.18790; *p* = 0.0303), and tumor necrosis factor‐alpha (TNF‐α: *r* = 0.18560; *p* = 0.0293) (Figure 3a). Markers known to decline with aging were also observed to have a negative correlation, including, transforming growth factor‐beta‐1 (TGF‐β‐1: *r* = −0.2012; *p* = 0.0202) and transforming growth factor‐beta‐2 (TGF‐β‐2: *r* = −0.2196; *p* = 0.0114) (Figure 3a). When segregated according to sex, GDF15 and MMP‐12 remained the most significant positively correlated proteins associated with chronological age (Figure 3b,c). Leptin, MMP‐3, and IP10 also remained statistically positively correlated with age. Interestingly females, lost the significant age‐associated positive correlations with SOST and negative correlation with TGF‐β, whereas it remained in these healthy men. When comparing these aging‐related serum protein signatures to levels of C_12_FDG^++^ PBMCs, similar strong positive and negative correlations were detected (Figure 3d). There were only two proteins that significantly correlated with senescence, but not age: parathyroid hormone (PTH, *r* = 0.2066; *p* = 0.0486) and MMP‐9 (*r* = 0.1921; *p* = 0.0444). Males specifically drove the correlation between C_12_FDG^++^ and MMP‐9 (Figure 3f), as this was not significantly correlated in females (Figure 3e). The other biomarkers found to positively correlate with increasing C_12_FDG^++^ signal include GDF‐15 (*r* = 0.3731; *p* = 0.0012), SOST (*r* = 0.2066; *p* = 0.0457), fibroblast growth factor‐23 (FGF23: *r* = 0.2018; *p* = 0.0537), and MMP‐3 (*r* = 0.1909; *p* = 0.0489). Interesting correlations between C_12_FDG^++^ appear to be sexually dimorphic with different proteins presenting as significant between the sexes, with only GDF15 being consistent across the sexes (Figure 3e,f). Biomarkers that uniquely correlated with C_12_FDG^++^ PBMCs in females were monocyte chemoattractant protein‐1 (MCP‐1: *r* = 0.4737; *p* = 0.0002), MMP‐3 (*r* = 0.3242; *p* = 0.0298), and platelet‐derived growth factor‐AA (PDGF‐AA: *r* = 0.3125; *p* = 0.0202); TGF‐β‐1 (*r* = −0.2696; *p* = 0.0487) and TGF‐β‐2 (*r* = −0.3140; *p* = 0.0208) were negatively correlated in females (Figure 3e). Biomarkers that uniquely correlated with C_12_FDG^++^ PBMCs in males were MMP‐1 (*r* = 0.3949; *p* = 0.0020), MMP‐9 (*r* = 0.2263; *p* = 0.0250), and MMP‐12 (*r* = 0.2763; *p* = 0.0297) (Figure 3f). These data support that there is a high degree of overlap between the secretome associated with aging and the percent of C_12_FDG^++^ PBMCs. Meanwhile, there is not a high degree of overlap of biomarkers with both aging and the percent C_12_FDG^++^ PBMCs with the percent C_12_FDG^++^ TCs. Biomarkers found to positively correlate with the percent C_12_FDG^++^ TCs include RANTES (*r* = 0.3102; *p* = 0.0002) across both sexes, MMP‐10 (*r* = 0.2825; *p* = 0.0020), MMP‐9 (*r* = 0.2439; *p* = 0.0070), and MMP‐1 (*r* = 0.2192; *p* = 0.0203); in addition, growth regulated oncogene (GRO: *r* = −0.2655; *p* = 0.0015) is found to negatively correlate with the percent C_12_FDG^++^ TCs in males only (Figure 3g–i).

### 3.4 C_12_FDG ^++^ PBMCs and senescence‐associated serum biomarkers are reduced in patients taking fisetin

**FIGURE 4:**
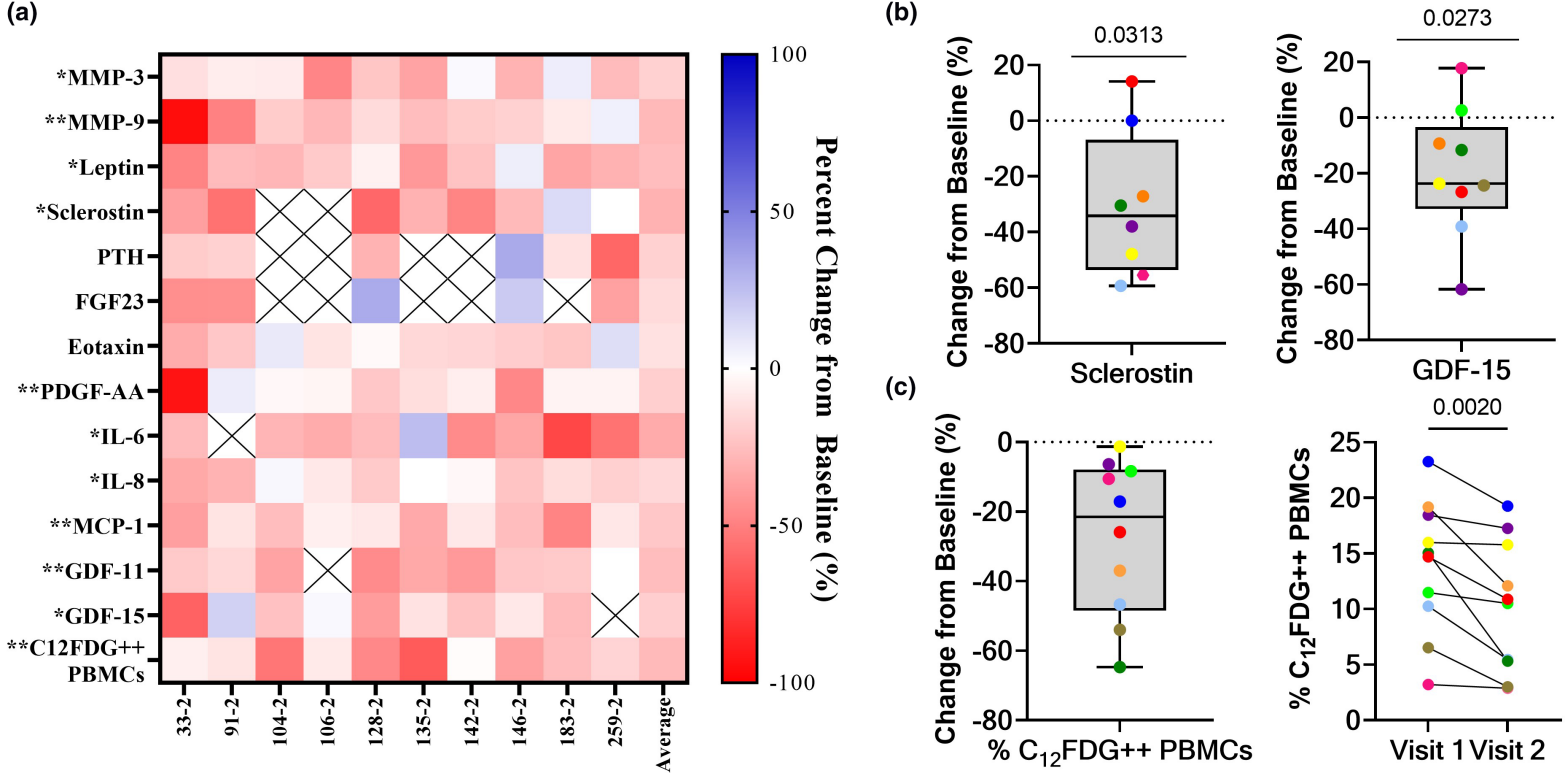
Longitudinal reduction of C_12_FDG^++^ PBMCs and associated serum biomarkers in patients reporting taking fisetin. (a) Heat map of percent change in aging and senescence‐associated serum biomarkers and % C_12_FDG^++^ (bright) PBMCs in patients reported taking fisetin compared to baseline (*n* = 10; days on drug = 58 ± 31; age = 70 ± 13 years; 6 M/4F). Significant differences are denoted by * (<0.05) and ** (<0.01). (b) Percent change from baseline of the bone inhibitor sclerostin (left) and the aging marker GDF15 (right) in participants reporting taking the senolytic drug fisetin (100 mg/day) compared to baseline (no fisetin) between two visits (*n* = 8–9). (c) Percent change (left) and absolute change (right) of % C_12_FDG^++^ (bright) PBMCs in participants following fisetin usage (*n* = 10). Significance determined using Wilcoxon matched pairs signed rank testing. Comprehensive summary of all changes in analytes not correlated with C_12_FDG^++^ PBMCs are listed in Figure S3.

Fisetin is a senolytic flavonoid that can extend healthspan in naturally aged and progeroid mice (Yousefzadeh et al., 2018). Medical histories were collected from study participants prior to blood collection and a subset of 10 participants reported taking 100 mg/day of fisetin between baseline and subsequent study visits. Participants taking other doses of fisetin or other senotherapeutic compounds were excluded from this analysis. To examine the effects of fisetin on peripheral blood‐related senescence signatures, samples from participants were analyzed longitudinally prior to and following reported fisetin treatment. When comparing serum biomarkers related to aging/senescence after fisetin usage, MMP‐3, MMP‐9, leptin, SOST, PDGF‐AA, IL‐6, IL‐8, MCP‐1, GDF‐11, and GDF‐15 were all found to be significantly decreased (Figure 4a, Figure S3). The two biomarkers of particular interest were GDF‐15 and SOST (Figure 4b) as they were shown to strongly correlate with both age and C_12_FDG^++^ PBMCs (Figure 3a,b). Furthermore, the percentage of bright C_12_FDG^++^ PBMCs was also found to be significantly reduced by an average of 27.2%, SD = 22.203 (*p* = 0.0020) in participants who reported taking fisetin (Figure 4c). C_12_FDG^++^ quantification in PBMCs thus shows sensitivity to senolytic treatment with fisetin.

### 3.5 C_12_FDG ^++^ and senescence biomarkers are elevated in patients with osteoarthritis

**FIGURE 5:**
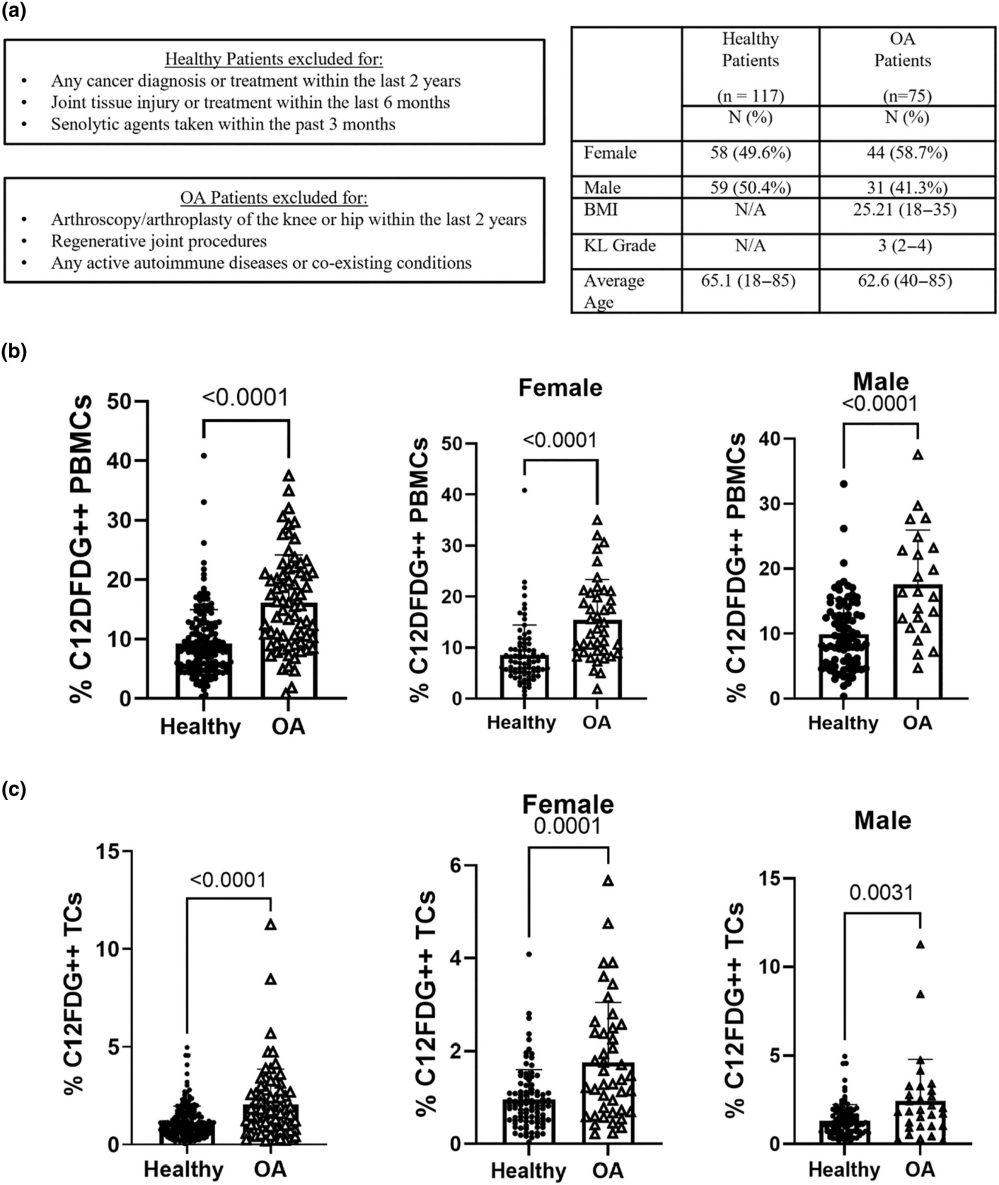
Increased C_12_FDG signal in patients with mild to moderate OA. (a) Schematic of patient selection criteria and demographics for healthy patients (*n* = 117 patients) and OA patients (*n* = 75 patients). (b) Percent C_12_FDG^++^ PBMCs in healthy compared to OA patients. (Healthy *n* = 168 visits; OA *n* = 66. Female Healthy *n* = 81; Female OA *n* = 43. Male Healthy *n* = 87; Male OA *n* = 23). (c) Percent C_12_FDG^++^ T cells in healthy compared to OA patients. (Healthy *n* = 204 visits; OA *n* = 75. Female Healthy *n* = 98; Female OA *n* = 44. Male Healthy *n* = 106; Male OA *n* = 31). Results were analyzed using a Mann–Whitney test.

Knee osteoarthritis (OA) is associated with the overproduction of pro‐inflammatory and cartilage‐degrading factors from resident and infiltrating cells (Loeser et al., 2012; Robinson et al., 2016). To determine if peripheral blood C_12_FDG quantification increases with OA, C_12_FDG^++^ PBMCs and T cells were analyzed in patients with mild to moderate OA compared to asymptomatic healthy controls in a cross‐sectional analysis of two ongoing studies at our site. The percent of C_12_FDG^++^ cells was significantly elevated in OA versus healthy patients in both PBMCs (Figure 5b) and T cells (Figure 5c). The statistically significant increase in C_12_FDG^++^ also was conserved when the sample population was separated by sex. Gene expression analysis from a subset of these patients supports a senescent‐like phenotype with significantly elevated *p21* expression (Figure S4).

## 4 DISCUSSION

Senescent cells and their senescence‐associated secretory phenotype (SASP) are known to promote inflammation and many age‐associated diseases such as diabetes, cardiovascular diseases, and neurodegeneration (Gorgoulis et al., 2019). Cellular senescence is a fundamental mechanism by which cells remain metabolically active, but cease dividing and undergo distinct phenotypic changes, including upregulation of *p16* ^*Ink4a*^ (*p16*), significant secretome changes, telomere shortening, and decompensation of pericentromeric satellite DNA (Gorgoulis et al., 2019). Expression of *p16* is significantly increased in B cells, T cells, myeloid cells, osteoblast progenitor cells, osteoblasts, and osteocytes in naturally aged mice as well as a variety of non‐lymphoid organs (Farr et al., 2016). A senescence‐like state of immune cells has been linked to the increased chronic inflammation that can occur during aging, termed both immunosenescence and inflammaging. Inflammaging is associated with a reduced ability to respond to new antigens, due to decreases in naïve T‐ and B cells, with an increased abundance of memory T cells (Pawelec, 2018). Due to thymic involution with aging, T cells in particular demonstrate an increase in CD8/CD4 ratio and exhibit senescence phenotypes, including telomere shortening, increased expression of the programmed death proteins (PD‐1) and CD57, increased expression of pro‐inflammatory cytokines, and decreased expression of co‐stimulatory markers such as CD28 (Pawelec, 2018; Plunkett et al., 2007). However, the current repertoire of T‐cell markers to identify senescent phenotypes may not completely distinguish all senescent T‐cell subsets in vivo given reports of retained proliferation potential in T cells that fit the traditional cell‐surface marker criterion (Martinez‐Zamudio et al., 2021). Understanding senescence mechanisms and dynamic changes with aging in circulating immune cells is thus important to identify therapeutic strategies to improve immunological function with advanced age, including improving responsiveness to vaccines and tissue regeneration/repair mechanisms that are dependent on harmonized inflammatory cells and factors.

Senotherapeutics that interfere with and delay the aging processes have been demonstrated to target and modulate senescent cell and their SASP production (Gorgoulis et al., 2019; Kirkland et al., 2017). These include senolytics, which kill senescent cells, and senomorphics that modulate functions of senescent cells, inhibit (SASP) and reduce inflammation/fibrosis (Chaib et al., 2022; Kirkland et al., 2017; Xu et al., 2021; Yousefzadeh et al., 2018). Elimination of these senescent cells with senolytics can alleviate age‐related pathologies and extend healthspan in naturally aged and progeroid mice (Farr et al., 2017; Gorgoulis et al., 2019; Kirkland et al., 2017; Palmer et al., 2019; Xu et al., 2015, 2018; Yousefzadeh et al., 2018). Several clinical trials are now underway from our group and others investigating the therapeutic efficacy of senolytics in mitigating several age‐related morbidities (Chaib et al., 2022). Fisetin is a natural flavonoid found to have senolytic activity by targeting senescence‐associated pathways such as SIRT1, BCL‐2/BCL‐X_L_, HIF‐1α, p53/MDM2, and AKT, leading to elimination of senescent cells and reduction in SASP‐driven inflammation both in vitro and in vivo (Zhang & Jia, 2016).

Here, we validate a flow cytometry‐based assay to detect senescent‐like peripheral blood mononuclear cells (PBMCs), including analysis in the subset of T cells, using the fluorescent compound C_12_FDG. C_12_FDG (5‐Dodecanoylaminofluorescein Di‐β‐D‐Galactopyranoside) is a compound that when hydrolyzed by β‐galactosidase, an enzyme upregulated during senescence, fluoresces at a wavelength of 514 nm. We found that in PBMCs and enriched T cells, distinct populations of C_12_FDG^+^ “dim” and C_12_FDG^++^ “bright” cells occurred. C_12_FDG^++^ cells significantly correlated with increasing chronological age, exhibited co‐localization with CD87 (a senescence‐associated cell‐surface markers), and were reduced in a small subgroup of study participants taking the senolytic drug fisetin. Interestingly, C_12_FDG^+^ (dim) cells did not consistently associate with chronological age or responsiveness to fisetin usage. This phenomenon of *bright* and *dim* SA‐β‐Gal associated fluorogenic signal has been observed in at least one other study for T cells isolated from human peripheral blood (Martinez‐Zamudio et al., 2021). Here, the authors reported bright populations of CD8^+^ T cells, not CD4^+^ T cells, measured using SPiDER‐βGal were associated with chronological age. The clinical and/or pathological relevance of C_12_FDG^+^ thus remains unclear, however, emerging evidence suggests that senescent phenotypes are dynamic, cell‐type specific, and fall on a spectrum with early to late‐stage characteristics (Nakao et al., 2020). It is now generally thought that the senescence program can be divided to 4 distinguishable phenotypic states of initiation, early, full, and late‐stage senescence (Nakao et al., 2020). Early stages are distinguished by cell enlargement with an upregulation of TGF‐β followed by epigenetic alterations and senescence‐associated heterochromatin foci formation, resulting in significant metabolic shifts and potent production of SASP factors in later stages (Nakao et al., 2020). Our current data from human PBMCs demonstrated the ability to detect distinct populations of *bright* C_12_FDG^++^ cells and *dim* C_12_FDG^+^ cells, which we hypothesize may represent cells in different stages of senescence.

The correlation of *bright* C_12_FDG^++^ cells with extracellular biomarkers of age also provides strong supportive evidence that this assay may be a clinically relevant method to detect cells with functional senescence. One biomarker of particular interest was GDF‐15, a stress and inflammation responsive member of the TGF‐β superfamily that has been found to be a putative biomarker for aging (Conte et al., 2020; Liu et al., 2021; Pence et al., 2021). In our subjects, we found GDF‐15 to be strongly correlated with both chronological age and with percent C_12_FDG^++^ PBMCs. At a more functional level, we found that sclerostin, a glycoprotein secreted by osteoblasts and associated with the inhibition of bone formation, was also significantly associated with both chronological age and C_12_FDG^++^ PBMC. This correlation is important as senescent cells have been linked to osteoporosis (Farr & Khosla, 2019) and targeting senescence has been shown to significantly prevent cortical and trabecular bone resorption in murine models of osteoporosis (Farr et al., 2017). The decrease in bone mineral density with age is well‐documented and the CDC estimates that over 55 million people in the United States have osteoporosis. Here we show in a small number of patients taking 100 mg/day of fisetin that sclerostin expression was significantly reduced, indicating that certain senolytics may also improve bone health in a human population.

Accumulation of senescent cells is also known to be important in the pathogenesis of OA (Liu et al., 2022). Using the C_12_FDG staining we found significantly elevated C_12_FDG^++^ PBMCs and T cells in patients with OA compared to healthy subjects. Additionally, we found a significant correlation between C_12_FDG^++^ T cells and known OA serum biomarkers. While less association was found between C_12_FDG^++^ PBMCs and OA serum biomarkers, positive correlations were observed with CRP, a well‐characterized prognostic marker for OA (Saberi Hosnijeh et al., 2016). Differences between senescent T‐cell and PBMC correlations with biomarkers such as CRP suggest that percent of senescent‐like PBMCs and T cells might both serve as unique indicators reflective of OA severity or symptomology. For example, increased CRP is best correlated with pain and not radiographic grade (Alexander et al., 2021). These data highlight the potential diagnostic utility of tracking senescence in peripheral blood during osteoarthritic sequelae, especially considering senescence is a known contributor to OA (Xu et al., 2017).

Further, the ability to track senescent cell indices systemically will allow for the evaluation of efficacy for treatments targeting senescence, an increasingly common interventional strategy for age‐related diseases. Overall, these data suggest that senescent PBMCs, namely T cells, may be predictive of OA using an established detection methodology that is rapid, technically simple, and economical. This study was limited by only evaluating C_12_FDG signatures in overall PBMCs and total T cells that were isolated through enrichment rather than cell‐specific antibodies due to our intended goal of developing a technically easy and rapid approach to measuring systemic senescence in a clinical setting. The predicted bias in CD4^+^ versus CD8^+^ senescence intensity with age noted by Martinez‐Zamudio et al. may also explain why we found significant increases in C_12_FDG intensity with age in only total PBMCs and not total (CD3^+^) T cells (Martinez‐Zamudio et al., 2021). Subsequent prospective immunophenotyping of specific myeloid and lymphoid cell populations could provide additional insight into the cellular and mechanistic drivers of senescence.

Taken together, these data provide key evidence for the clinical utility and sensitivity of the C_12_FDG stain in detecting and interrogating peripheral senescence. Our findings also support the likelihood that fisetin can reduce functional senescence in humans and have the potential to alleviate multiple age‐related morbidities through the reduction of immunosenescence. However, follow‐up analysis is needed to determine whether the benefits of fisetin are sustained and how they change over time. A randomized controlled trial is currently underway at our clinic testing the efficacy of fisetin in reducing mild to moderate OA symptomology with diverse outcome measures that when unblinded, will certainly highlight protective effects of senolytic therapy in OA. Future controlled trials to determine the optimum senolytic dosing regimen (fisetin or otherwise), gender effects, cell‐specific effects, and/or SASP modulation will add to our understanding of senotherapeutic efficacy in various health conditions. The C_12_FDG platform may thus provide additional quantifiable clinical tools to assess healthy aging and also serve as a potential indicator of acute and chronic diseases associated with aging.

## AUTHOR CONTRIBUTIONS

Conceptualization: WSH, JH – Lead; CSB, JLK, TT, JMEN, PDR, LJN, MJP – Supporting. Data Curation: VD, ADG, WSH, CSB. Formal Analysis: WSH, CSB, Lead; ADG, HK, AWG, LTM – Supporting. Funding Acquisition: JH, MJP – Lead; WSH – Supporting. Investigation: VD, CH, WSH, AWG, HK, XG, CH, PG, AL, JM, MM. Methodology: SH, JH – Lead; CSB, JLK, TT, JMEN, PDR, LJN, MJP – Supporting. Project Administration: CSB, WSH, JH. Resources: JH – Lead; MJP – Supporting. Supervision: WSH, CSB, JH. Validation: CSB, JH, WSH – Lead; VD, HK, AWG – Supporting. Visualization: VRD, WSH, ADG, AWG, HK, CSB, LTM. Writing – Original Draft Preparation: WSH, CSB, JH – Lead; CS – Supporting. Writing – Review & Editing: CSB, JH – Lead; PDR, LTM, CS, TT, JMEN, LJN, JLK – Support.

## FUNDING INFORMATION

Research reported in this publication was supported by the National Institute of Arthritis and Musculoskeletal and Skin Diseases (NIAMS) of the National Institutes of Health (NIH) under Award Number UH3AR077748 (Huard) and the Department of Defense Office of Naval Research under contract N00014‐19‐C‐2052 (Huard). The content is solely the responsibility of the authors and does not necessarily represent the official views of the National Institutes of Health or the Department of Defense. Major philanthropic support to the institute and this study were provided by Mitch & Linda Hart and the Borgen Foundation. Research was also aided by a grant form Orthopaedic Research and Education Foundation (OREF) with funding provided by The Aircast Foundation number TAF‐21‐059 (Bahney).

## CONFLICT OF INTEREST STATEMENT

Drs Hambright, Ravuri, Philippon and Huard declare inventorship on US PCT Application 16994356 **“**Methods for treating disease associated with senescence”. Dr. Bahney discloses IP royalties from Iota Biosciences, Inc. for US Patent 041263 and an Associate Editor role for the Journal of Tissue Engineering and Regenerative Medicine (JTERM).

## Supporting information


Appendix S1

## Data Availability

The data that support the findings of this study are available from the corresponding author upon reasonable request.
