# Supplementary material for: Clinical validation of C12FDG as a marker associated with senescence and osteoarthritic phenotypes
Source: Aging Cell. 2024 May 6;23(5):e14113. doi: 10.1111/acel.14113 (PMC11113632; doi:10.1111/acel.14113)
Supplement: Supplementary file 1 — Appendix S1 [file ACEL-23-e14113-s001.docx]

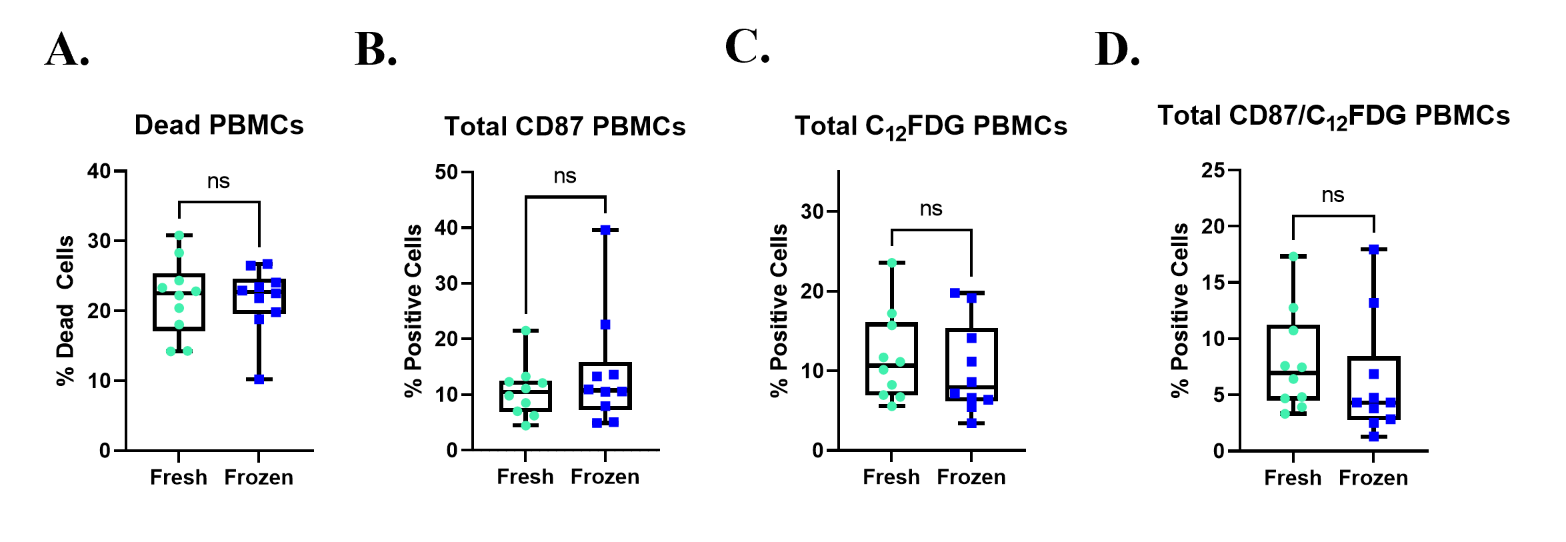
 **Supplemental Figure 1. Effects of freezing on CD87 and C_12_FDG staining.** **(A)** No significant change in the viability of cells between the fresh and frozen samples (p = 0.9406). **(B)** Percent positive cells in PBMCs ran fresh versus post-freezing for CD87 cells indicating no significant change in the populations by flow cytometry (p = 0.1537). **(C)** Percent positive cells in PBMCs ran fresh vs. post-freezing for total C_12_FDG cells (bright+dim) indicating no significant change in the populations by flow cytometry (p = 0.3086). **(D)** Percent positive cells in PBMCs ran fresh versus post-freezing for total CD87/C_12_FDG cells (bright+dim) indicating no significant change in the populations by flow cytometry (p = 0.1129).


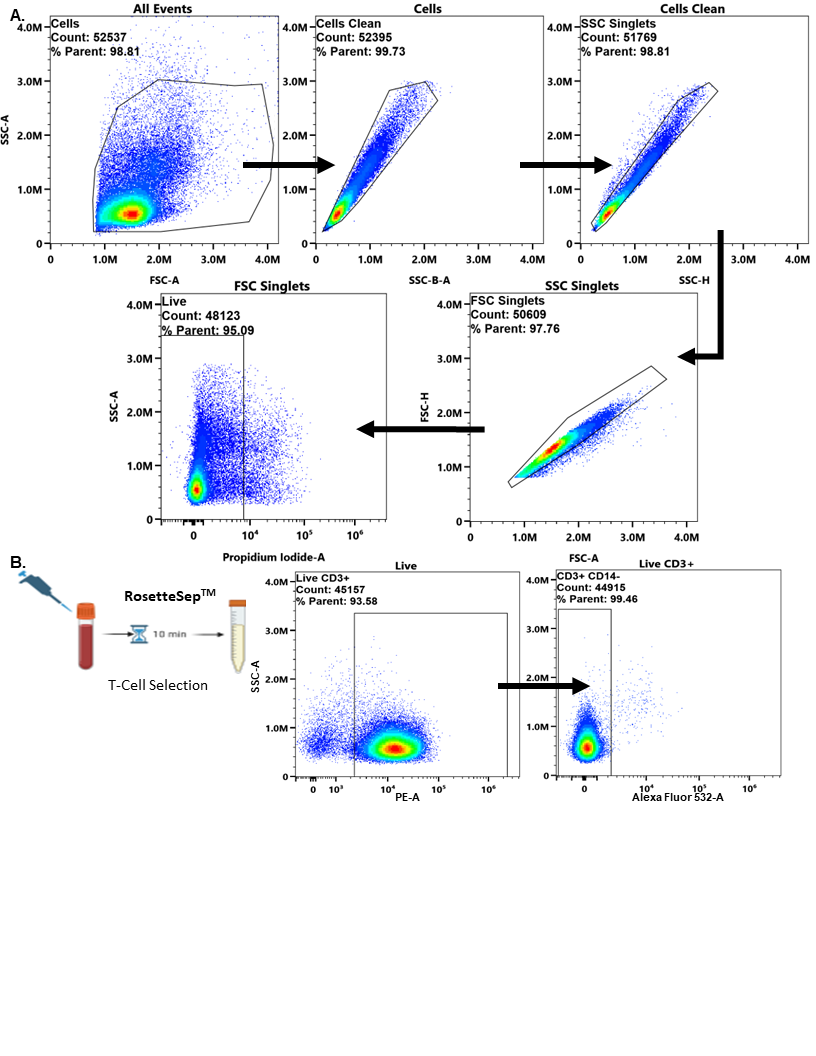


**Supplemental Figure 2. Gating strategy for detection of C_12_FDG^+^ PBMCs and PBMC subsets.** **(A)** Gating criterion for live cell detection and doublet exclusion. **(B)** Enrichment strategy for CD3^+^ T-cells using RosetteSep (StemCell Technologies) indicating approximately 93% recovery of CD3^+^/CD14^-^ T-cells.


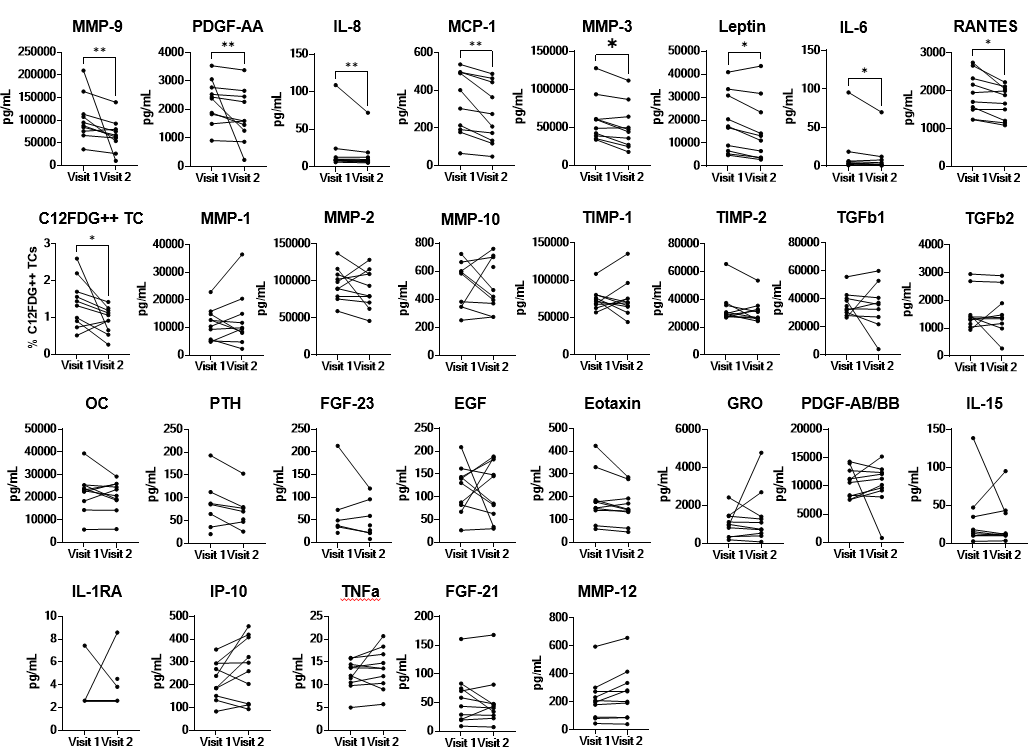


**Supplemental Figure 3.** Changes in concentration of aging and senescence serum biomarkers and C_12_FDG^++^ PBMCs and TCs in participants reporting taking the senolytic drug fisetin (100mg/day, visit 2) compared to baseline (no fisetin, visit 1). *Significance determined by Wilcoxon matched pairs signed rank testing.

**Supplemental Figure 4. Increased *p21* expression OA patients compared to healthy.** (A) *p21* expression measured by quantitative RT-PCR is significantly increased in OA patients (*Results were analyzed using a Mann-Whitney test, healthy n = 7, OA n = 14, p = 0.0001). T-cells were lysed for gene expression with TRIzol Reagent (Invitrogen) following enrichment using the RosetteStep^TM^ (**Supplemental Figure 3**) per manufactures standard protocol. cDNA was reverse transcribed using qScript cDNA synthesis kit (Quanta Bio) and then qRT-PCR run using standard SYBR Green Master Mix (ThermoFisher) using the *p21* primer sequences - Forward: 5’ CAA GCT CTA CCT TCC CAC GG 3’ Reverse: 5’ ATC TGT CAT GCT GGT CTG CC 3’.
